# New Methods in RNA Structural Biology: TELSAM Protein Chaperone Crystallography Applications to Folded RNA

**DOI:** 10.1063/4.0001119

**Published:** 2025-10-27

**Authors:** Jacob C Averett, Miles Bradford, Blake Averett, Dalton Hansen, James D Moody

**Affiliations:** Brigham Young University, Provo, Utah, USA

## Abstract

The multifaceted roles of RNA in cellular function are becoming increasingly apparent, and the importance of deepening our understanding of the function and structure of ribozymes and non-enzymatic RNAs will continue to grow. Comparatively few RNA structures have been solved, so it is evident that much more structural work needs to be done on these vital biological molecules. We present here a new technique in development that employs the crystallization chaperone properties of the recombinant TELSAM protein covalently attached to a target RNA in vitro to facilitate high resolution crystallography of folded RNA molecules. We designed the synthesis of a novel ribonucleic acid modification, 2- maleimidoacetyldideoxycytosine triphosphate, which can be incorporated into any position of an RNA molecule by direct synthesis methods, or on the 3’ end by enzymatic or non-templated polymerase action. We then designed TELSAM protein chaperone monomers with a c-terminal cysteine mutation, which will selectively react with the maleimide group on the modified RNA molecule below pH 7.5, assembling a hybrid protein-nucleic acid molecule that we should then be able to crystallize in a similar fashion to routine TELSAM-mediated protein crystallography. Our hope is that this technique will enable facile and inexpensive structural determination of folded RNA molecules and contribute to a significant deepening of our understanding of RNA biochemistry.

